# Ethyl 6-ethoxy­carbonyl­methyl-4-(2-hydroxy­phen­yl)-2-oxo-1,2,3,4-tetra­hydro­pyrimidine-5-carboxyl­ate

**DOI:** 10.1107/S1600536808012683

**Published:** 2008-05-17

**Authors:** Viktor Kettmann, Jan Světlík, Lucia Veizerová

**Affiliations:** a Faculty of Pharmacy, Comenius University, Odbojarov 10, SK-83232 Bratislava, Slovakia

## Abstract

The title compound, C_17_H_20_N_2_O_6_, belongs to the monastrol-type of anti­cancer agents and was selected for crystal structure determination in order to confirm its mol­ecular structure and explore some aspects of its structure–activity relationships. The central tetra­hydro­pyrimidine ring has a flat-envelope conformation. The 4-hydroxy­phenyl group occupies a pseudo-axial position and is inclined at an angle of 87.7 (2)° to the mean plane of the heterocyclic ring. Of the two ethyl ester groups, one (in the 5-position) is in a coplanar and the other (in the 6-position) is in a perpendicular orientation with respect to the heterocyclic plane. There is a three-dimensional hydrogen-bonding network in which all hydrogen-bond donors and acceptors are involved.

## Related literature

For related literature, see: Allen (2002 ▶); Azizian *et al.* (2007 ▶); Qing-Fang *et al.* (2007 ▶); Endow & Baker (2003 ▶); Kettmann & Svetlík (1997 ▶); Světlík *et al.* (1991 ▶); Wood & Bergnes (2004 ▶).
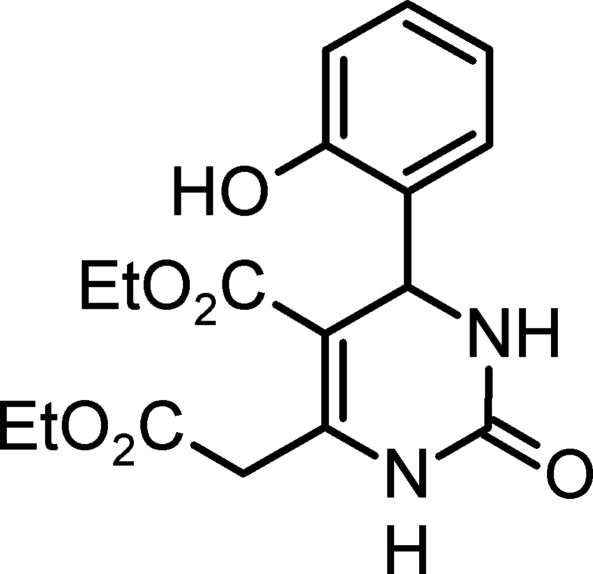


## Experimental

### 

#### Crystal data

C_17_H_20_N_2_O_6_
*M* *_r_* = 348.35
Triclinic, 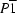

*a* = 8.783 (2) Å
*b* = 9.336 (3) Å
*c* = 11.415 (4) Å
α = 71.47 (4)°
β = 82.78 (5)°
γ = 75.05 (4)°
*V* = 856.5 (5) Å^3^
*Z* = 2
Mo *K*α radiation
μ = 0.10 mm^−1^
*T* = 296 (2) K
0.30 × 0.25 × 0.20 mm

#### Data collection

Siemens P4 diffractometer
Absorption correction: none
5849 measured reflections
4950 independent reflections
3935 reflections with *I* > 2σ(*I*)
*R*_int_ = 0.040
3 standard reflections every 97 reflections intensity decay: none

#### Refinement

*R*[*F* ^2^ > 2σ(*F* ^2^)] = 0.057
*wR*(*F* ^2^) = 0.174
*S* = 1.04
4950 reflections
229 parameters
H-atom parameters constrained
Δρ_max_ = 0.31 e Å^−3^
Δρ_min_ = −0.32 e Å^−3^

### 

Data collection: *XSCANS* (Siemens, 1991 ▶); cell refinement: *XSCANS*; data reduction: *XSCANS*; program(s) used to solve structure: *SHELXS97* (Sheldrick, 2008 ▶); program(s) used to refine structure: *SHELXL97* (Sheldrick, 2008 ▶); molecular graphics: *PLATON* (Spek, 2003 ▶); software used to prepare material for publication: *SHELXL97*.

## Supplementary Material

Crystal structure: contains datablocks global, I. DOI: 10.1107/S1600536808012683/wk2083sup1.cif

Structure factors: contains datablocks I. DOI: 10.1107/S1600536808012683/wk2083Isup2.hkl

Additional supplementary materials:  crystallographic information; 3D view; checkCIF report

## Figures and Tables

**Table 1:** Hydrogen-bond geometry (Å, °)

*D*—H⋯*A*	*D*—H	H⋯*A*	*D*⋯*A*	*D*—H⋯*A*
N1—H1⋯O1^i^	0.86	1.97	2.788 (2)	159
O2—H2⋯O3^ii^	0.82	1.95	2.772 (2)	177
N3—H3⋯O5^iii^	0.86	2.20	3.0014 (18)	155

Symmetry codes: (i) 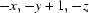; (ii) 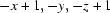; (iii) 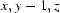.

**Fig. 1.**
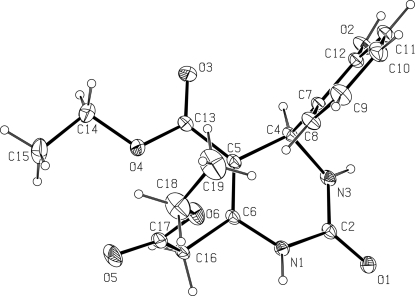
Displacement ellipsoid plot of (2) with the labelling scheme for the non-H atoms, which are drawn as 35% probability ellipsoids.

**Fig. 2.**
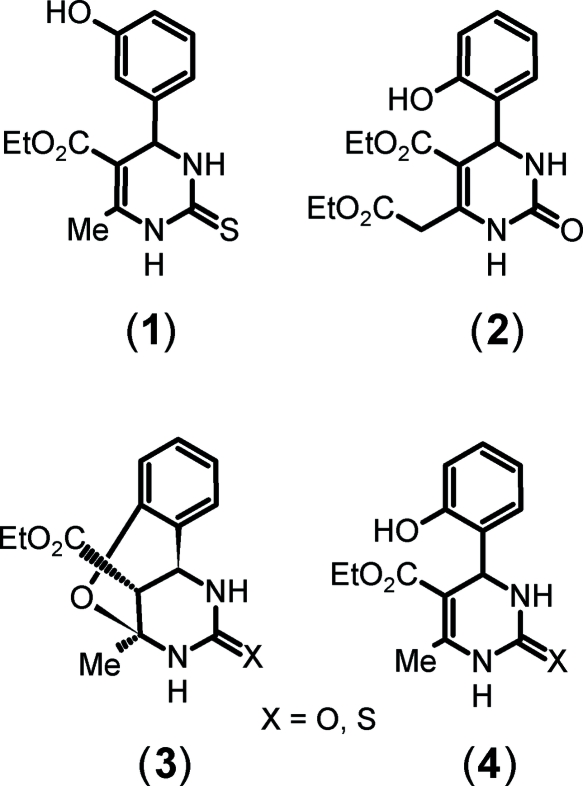
The structure of compounds (1)–(4).

**Fig. 3.**
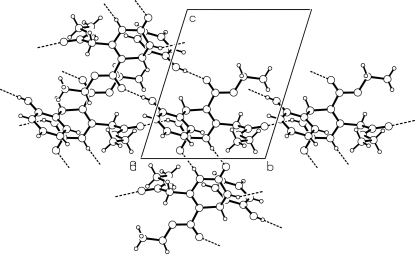
View of the crystal packing along the a axis, showing system of hydrogen bonds (dashed lines).

## supplementary crystallographic information

### Comment

Recently, while we have been continuing in our programme aimed at synthesis of monastrol (1) analogues as valuable antitumour drugs (Wood & Bergnes, 2004), a related tetrahydropyrimidine compound (2) has been described by another group (Azizian *et al.*, 2007). As we had reported earlier (Světlík *et al.*, 1991; Kettmann & Svetlík, 1997) that classical Biginelli condensation with salicylaldehyde gives oxygen-bridged pyrimidine (3) rather than the 'open' molecule (4), the formation of (2) was accordingly unexpected. Thus, to verify the correctness of the title structure (2), an X-ray analysis was undertaken. As the cytotoxic activity of these derivatives is related to inhibition of the kinesin Eg5 protein (Endow & Baker, 2003), another purpose of this work was to determine detailed molecular conformation which is indispensable for an analysis of structure-activity relationships.

The structure determination has confirmed (Fig. 1) that the compound studied here has indeed the structure (2) (Fig. 2). As retrieved from the Cambridge Structural Database (Version of 2007; Allen, 2002), the bond lengths and angles (Table 1) within the tetrahydropyrimidine ring are equal within experimental error to those previously reported for a number of structures incorporating this molecular fragment (see, *e.g.*, Qing-Fang *et al.*, 2007). Bonding characteristics in other parts of the molecule also agree with those generally expected.

As noted above, from the biological standpoint, the conformational properties of the molecule are of prime interest here. First, the conformation of the central heterocycle can best be described as a flat envelope with atom C4 (at the flap) deviating by 0.433 (2) Å from the mean plane of the remaining atoms. As to the ring substituents, the 4-hydroxyphenyl group occupies the pseudoaxial position and is in a perpendicular orientation with respect to the tetrahydropyrimidine ring [dihedral angle 87.7 (2)°]; the conformation of the substituent on the exocyclic C4—C7 bond is synperiplanar, *i.e.* the hydroxy group is on the same side as the H atom on C4 (Fig.1). The ester group on C5 lies approximately in the plane of the C5=C6 double bond, with the carbonyl function oriented *cis* relative to this double bond. By contrast, the ethoxycarbonyl moiety of the 6-substituent is oriented, due to rather free rotation about the two C16 methylenic bonds, perpendicularly with respect to the mean plane of the heterocycle.

The crystal packing is dominated by hydrogen bonding. As shown in Table 2 and Fig. 3, each molecule forms two pairs of hydrogen bonds (N1-H···O1 and O2-H···O3) across centres of symmetry, which results in formation of chains of hydrogen-bonded molecules. The chains are interconnected by another independent hydrogen bond, N3-H···O5.

### Experimental

Synthesis of the title compound, (2), has been described (Azizian *et al.*, 2007). In short, heating of salicylaldehyde (0.52 ml, 5 mmol) with diethyl acetone-1,3-dicarboxylate (0.91 ml, 5 mmol) and urea (0.36 g, 6 mmol) under *p*-toluenesulfonic acid (0.04 g, 0.2 mmol) catalysis without solvent at 353–363 K for 3 h gave the desired product (50% yield; m.p. 488–490 K). Crystalssuitable for the X-ray analysis were obtained by a slow crystallization from ethanol.

### Refinement

H atoms were visible in difference maps and were subsequently treated as riding atoms with distances C—H = 0.93 Å (CH_arom_), 0.97 (CH_2_) or 0.98 Å (CH), 0.96 Å (CH_3_) and N—H = 0.86 Å and O—H = 0.82 Å; *U*_iso_ of the H atoms were set to 1.2 (1.5 for the methyl and hydroxy H atoms) times *U*_eq_ of the parent atom.

### Crystal data

**Table:**

C_17_H_20_N_2_O_6_	*Z* = 2
*M**_r_* = 348.35	*F*_000_ = 368
Triclinic, *P*1	*D*_x_ = 1.351 Mg m^−^^3^
Hall symbol: -P 1	Melting point: 489 K
*a* = 8.783 (2) Å	Mo *K*α radiation λ = 0.71073 Å
*b* = 9.336 (3) Å	Cell parameters from 20 reflections
*c* = 11.415 (4) Å	θ = 7–18º
α = 71.47 (4)º	µ = 0.10 mm^−^^1^
β = 82.78 (5)º	*T* = 296 (2) K
γ = 75.05 (4)º	Prism, colourless
*V* = 856.5 (5) Å^3^	0.30 × 0.25 × 0.20 mm

### Data collection

**Table:**

Siemens P4 diffractometer	*R*_int_ = 0.040
Radiation source: fine-focus sealed tube	θ_max_ = 30.0º
Monochromator: graphite	θ_min_ = 1.9º
*T* = 296(2) K	*h* = −1→12
ω/2θ scans	*k* = −12→12
Absorption correction: none	*l* = −16→16
5849 measured reflections	3 standard reflections
4950 independent reflections	every 97 reflections
3935 reflections with *I* > 2σ(*I*)	intensity decay: none

### Refinement

**Table:**

Refinement on *F*^2^	Secondary atom site location: difference Fourier map
Least-squares matrix: full	Hydrogen site location: inferred from neighbouring sites
*R*[*F*^2^ > 2σ(*F*^2^)] = 0.057	H-atom parameters constrained
*wR*(*F*^2^) = 0.174	*w* = 1/[σ^2^(*F*_o_^2^) + (0.0972*P*)^2^ + 0.1877*P*] where *P* = (*F*_o_^2^ + 2*F*_c_^2^)/3
*S* = 1.04	(Δ/σ)_max_ = 0.001
4950 reflections	Δρ_max_ = 0.31 e Å^−^^3^
229 parameters	Δρ_min_ = −0.31 e Å^−^^3^
Primary atom site location: structure-invariant direct methods	Extinction correction: none

### Special details

**Table:**

Geometry. All e.s.d.'s (except the e.s.d. in the dihedral angle between two l.s. planes) are estimated using the full covariance matrix. The cell e.s.d.'s are taken into account individually in the estimation of e.s.d.'s in distances, angles and torsion angles; correlations between e.s.d.'s in cell parameters are only used when they are defined by crystal symmetry. An approximate (isotropic) treatment of cell e.s.d.'s is used for estimating e.s.d.'s involving l.s. planes.
Refinement. Refinement of *F*^2^ against ALL reflections. The weighted *R*-factor *wR* and goodness of fit *S* are based on *F*^2^, conventional *R*-factors *R* are based on *F*, with *F* set to zero for negative *F*^2^. The threshold expression of *F*^2^ > σ(*F*^2^) is used only for calculating *R*-factors(gt) *etc*. and is not relevant to the choice of reflections for refinement. *R*-factors based on *F*^2^ are statistically about twice as large as those based on *F*, and *R*- factors based on ALL data will be even larger.

### Fractional atomic coordinates and isotropic or equivalent isotropic displacement parameters (Å^2^)

**Table:**

	*x*	*y*	*z*	*U*_iso_*/*U*_eq_	
N1	0.10214 (15)	0.47412 (12)	0.13671 (11)	0.0350 (3)	
H1	0.0749	0.5465	0.0692	0.042*	
C2	0.11515 (15)	0.32189 (14)	0.13967 (13)	0.0315 (3)	
O1	0.06497 (13)	0.29448 (11)	0.05495 (10)	0.0408 (3)	
N3	0.17844 (14)	0.21317 (12)	0.24052 (11)	0.0344 (3)	
H3	0.1615	0.1218	0.2584	0.041*	
C4	0.27587 (15)	0.24302 (13)	0.32230 (12)	0.0304 (3)	
H4	0.2669	0.1704	0.4051	0.037*	
C5	0.21064 (15)	0.40660 (14)	0.33107 (12)	0.0307 (3)	
C6	0.13046 (15)	0.51589 (13)	0.23577 (12)	0.0302 (3)	
C7	0.44794 (15)	0.21163 (14)	0.27695 (12)	0.0315 (3)	
C8	0.51674 (18)	0.32744 (17)	0.19754 (16)	0.0431 (3)	
H8	0.4584	0.4297	0.1753	0.052*	
C9	0.6717 (2)	0.2929 (2)	0.15072 (18)	0.0521 (4)	
H9	0.7161	0.3715	0.0975	0.063*	
C10	0.75860 (19)	0.1417 (2)	0.18372 (18)	0.0519 (4)	
H10	0.8617	0.1182	0.1519	0.062*	
C11	0.69370 (19)	0.02481 (19)	0.26383 (17)	0.0469 (4)	
H11	0.7536	−0.0768	0.2867	0.056*	
C12	0.53881 (16)	0.05890 (15)	0.31031 (14)	0.0353 (3)	
O2	0.46665 (14)	−0.05252 (12)	0.38635 (12)	0.0489 (3)	
H2	0.5328	−0.1341	0.4106	0.073*	
C13	0.24784 (17)	0.43413 (15)	0.44190 (13)	0.0367 (3)	
O3	0.31840 (19)	0.33335 (14)	0.52678 (12)	0.0602 (4)	
O4	0.19773 (16)	0.58055 (12)	0.44378 (11)	0.0485 (3)	
C14	0.2312 (3)	0.6194 (2)	0.55027 (18)	0.0582 (5)	
H14A	0.3441	0.6037	0.5551	0.070*	
H14B	0.1912	0.5542	0.6260	0.070*	
C15	0.1516 (3)	0.7858 (2)	0.5332 (2)	0.0668 (6)	
H15A	0.1992	0.8497	0.4624	0.100*	
H15B	0.1627	0.8131	0.6055	0.100*	
H15C	0.0417	0.8016	0.5208	0.100*	
C16	0.06044 (17)	0.68574 (14)	0.22359 (14)	0.0363 (3)	
H16A	−0.0109	0.7274	0.1562	0.044*	
H16B	−0.0022	0.6925	0.2988	0.044*	
C17	0.17511 (19)	0.78803 (15)	0.20076 (15)	0.0403 (3)	
O5	0.13786 (18)	0.91436 (13)	0.21651 (16)	0.0651 (4)	
O6	0.31602 (15)	0.72710 (13)	0.15956 (13)	0.0521 (3)	
C18	0.4286 (3)	0.8264 (3)	0.1244 (3)	0.0778 (7)	
H18A	0.4017	0.9049	0.0460	0.093*	
H18B	0.4240	0.8786	0.1864	0.093*	
C19	0.5842 (3)	0.7343 (3)	0.1139 (4)	0.0954 (9)	
H19A	0.6129	0.6613	0.1931	0.143*	
H19B	0.6572	0.8004	0.0864	0.143*	
H19C	0.5870	0.6792	0.0553	0.143*	

### Atomic displacement parameters (Å^2^)

**Table:**

	*U*^11^	*U*^22^	*U*^33^	*U*^12^	*U*^13^	*U*^23^
N1	0.0449 (6)	0.0191 (5)	0.0410 (6)	−0.0060 (4)	−0.0108 (5)	−0.0068 (4)
C2	0.0302 (6)	0.0220 (5)	0.0430 (7)	−0.0070 (4)	−0.0017 (5)	−0.0100 (5)
O1	0.0490 (6)	0.0278 (5)	0.0503 (6)	−0.0086 (4)	−0.0120 (5)	−0.0145 (4)
N3	0.0371 (6)	0.0183 (4)	0.0485 (6)	−0.0078 (4)	−0.0076 (5)	−0.0076 (4)
C4	0.0316 (6)	0.0192 (5)	0.0374 (6)	−0.0034 (4)	−0.0032 (5)	−0.0057 (4)
C5	0.0309 (6)	0.0208 (5)	0.0386 (6)	−0.0023 (4)	−0.0031 (5)	−0.0086 (4)
C6	0.0301 (6)	0.0200 (5)	0.0400 (6)	−0.0040 (4)	−0.0043 (5)	−0.0086 (4)
C7	0.0306 (6)	0.0246 (5)	0.0377 (6)	−0.0014 (4)	−0.0050 (5)	−0.0098 (5)
C8	0.0376 (7)	0.0299 (6)	0.0546 (9)	−0.0049 (5)	0.0012 (6)	−0.0062 (6)
C9	0.0421 (8)	0.0477 (9)	0.0610 (10)	−0.0127 (7)	0.0082 (7)	−0.0108 (7)
C10	0.0331 (7)	0.0566 (10)	0.0660 (11)	−0.0028 (7)	0.0024 (7)	−0.0262 (8)
C11	0.0364 (7)	0.0375 (7)	0.0644 (10)	0.0053 (6)	−0.0075 (7)	−0.0210 (7)
C12	0.0345 (6)	0.0257 (6)	0.0450 (7)	−0.0006 (5)	−0.0095 (5)	−0.0118 (5)
O2	0.0458 (6)	0.0227 (5)	0.0677 (8)	−0.0004 (4)	−0.0063 (5)	−0.0039 (5)
C13	0.0402 (7)	0.0268 (6)	0.0403 (7)	−0.0018 (5)	−0.0062 (5)	−0.0094 (5)
O3	0.0838 (10)	0.0372 (6)	0.0517 (7)	0.0105 (6)	−0.0301 (7)	−0.0116 (5)
O4	0.0702 (8)	0.0307 (5)	0.0453 (6)	0.0010 (5)	−0.0188 (5)	−0.0164 (4)
C14	0.0785 (13)	0.0520 (10)	0.0517 (10)	−0.0078 (9)	−0.0171 (9)	−0.0264 (8)
C15	0.0932 (16)	0.0557 (11)	0.0642 (12)	−0.0218 (11)	0.0058 (11)	−0.0351 (10)
C16	0.0368 (6)	0.0207 (5)	0.0507 (8)	0.0016 (5)	−0.0126 (6)	−0.0121 (5)
C17	0.0508 (8)	0.0217 (5)	0.0483 (8)	−0.0047 (5)	−0.0154 (6)	−0.0082 (5)
O5	0.0715 (9)	0.0265 (5)	0.1037 (11)	−0.0072 (5)	−0.0159 (8)	−0.0272 (6)
O6	0.0534 (7)	0.0329 (5)	0.0741 (8)	−0.0180 (5)	0.0064 (6)	−0.0185 (5)
C18	0.0722 (14)	0.0501 (11)	0.116 (2)	−0.0352 (10)	0.0059 (13)	−0.0184 (12)
C19	0.0650 (14)	0.0790 (17)	0.154 (3)	−0.0359 (13)	0.0172 (16)	−0.0439 (18)

### Geometric parameters (Å, °)

**Table:**

N1—C6	1.3761 (18)	C12—O2	1.366 (2)
N1—C2	1.3859 (16)	O2—H2	0.8200
N1—H1	0.8600	C13—O3	1.2161 (19)
C2—O1	1.2319 (17)	C13—O4	1.3299 (17)
C2—N3	1.3385 (19)	O4—C14	1.456 (2)
N3—C4	1.4733 (18)	C14—C15	1.490 (3)
N3—H3	0.8600	C14—H14A	0.9700
C4—C5	1.5172 (17)	C14—H14B	0.9700
C4—C7	1.5196 (19)	C15—H15A	0.9600
C4—H4	0.9800	C15—H15B	0.9600
C5—C6	1.3553 (19)	C15—H15C	0.9600
C5—C13	1.458 (2)	C16—C17	1.505 (2)
C6—C16	1.5134 (17)	C16—H16A	0.9700
C7—C8	1.390 (2)	C16—H16B	0.9700
C7—C12	1.3997 (18)	C17—O5	1.2047 (18)
C8—C9	1.393 (2)	C17—O6	1.318 (2)
C8—H8	0.9300	O6—C18	1.461 (2)
C9—C10	1.376 (3)	C18—C19	1.428 (4)
C9—H9	0.9300	C18—H18A	0.9700
C10—C11	1.381 (3)	C18—H18B	0.9700
C10—H10	0.9300	C19—H19A	0.9600
C11—C12	1.391 (2)	C19—H19B	0.9600
C11—H11	0.9300	C19—H19C	0.9600
			
C6—N1—C2	123.64 (12)	C12—O2—H2	109.5
C6—N1—H1	118.2	O3—C13—O4	121.72 (14)
C2—N1—H1	118.2	O3—C13—C5	123.83 (13)
O1—C2—N3	124.46 (12)	O4—C13—C5	114.46 (12)
O1—C2—N1	119.97 (13)	C13—O4—C14	118.33 (13)
N3—C2—N1	115.52 (12)	O4—C14—C15	107.06 (16)
C2—N3—C4	122.97 (11)	O4—C14—H14A	110.3
C2—N3—H3	118.5	C15—C14—H14A	110.3
C4—N3—H3	118.5	O4—C14—H14B	110.3
N3—C4—C5	109.07 (11)	C15—C14—H14B	110.3
N3—C4—C7	109.88 (11)	H14A—C14—H14B	108.6
C5—C4—C7	113.81 (11)	C14—C15—H15A	109.5
N3—C4—H4	108.0	C14—C15—H15B	109.5
C5—C4—H4	108.0	H15A—C15—H15B	109.5
C7—C4—H4	108.0	C14—C15—H15C	109.5
C6—C5—C13	125.23 (12)	H15A—C15—H15C	109.5
C6—C5—C4	119.05 (12)	H15B—C15—H15C	109.5
C13—C5—C4	115.65 (11)	C17—C16—C6	116.62 (12)
C5—C6—N1	119.39 (11)	C17—C16—H16A	108.1
C5—C6—C16	127.74 (12)	C6—C16—H16A	108.1
N1—C6—C16	112.86 (11)	C17—C16—H16B	108.1
C8—C7—C12	118.38 (13)	C6—C16—H16B	108.1
C8—C7—C4	122.55 (12)	H16A—C16—H16B	107.3
C12—C7—C4	118.97 (12)	O5—C17—O6	124.41 (16)
C7—C8—C9	121.11 (15)	O5—C17—C16	122.19 (16)
C7—C8—H8	119.4	O6—C17—C16	113.38 (12)
C9—C8—H8	119.4	C17—O6—C18	116.16 (15)
C10—C9—C8	119.64 (16)	C19—C18—O6	109.59 (19)
C10—C9—H9	120.2	C19—C18—H18A	109.8
C8—C9—H9	120.2	O6—C18—H18A	109.8
C9—C10—C11	120.39 (15)	C19—C18—H18B	109.8
C9—C10—H10	119.8	O6—C18—H18B	109.8
C11—C10—H10	119.8	H18A—C18—H18B	108.2
C10—C11—C12	120.09 (15)	C18—C19—H19A	109.5
C10—C11—H11	120.0	C18—C19—H19B	109.5
C12—C11—H11	120.0	H19A—C19—H19B	109.5
O2—C12—C11	122.69 (13)	C18—C19—H19C	109.5
O2—C12—C7	116.89 (13)	H19A—C19—H19C	109.5
C11—C12—C7	120.39 (15)	H19B—C19—H19C	109.5
			
C6—N1—C2—O1	168.78 (13)	C8—C9—C10—C11	−0.7 (3)
C6—N1—C2—N3	−8.8 (2)	C9—C10—C11—C12	1.0 (3)
O1—C2—N3—C4	162.57 (13)	C10—C11—C12—O2	177.68 (15)
N1—C2—N3—C4	−19.99 (19)	C10—C11—C12—C7	−0.3 (2)
C2—N3—C4—C5	36.47 (17)	C8—C7—C12—O2	−178.66 (14)
C2—N3—C4—C7	−88.92 (15)	C4—C7—C12—O2	−2.32 (19)
N3—C4—C5—C6	−27.05 (17)	C8—C7—C12—C11	−0.5 (2)
C7—C4—C5—C6	96.02 (15)	C4—C7—C12—C11	175.82 (13)
N3—C4—C5—C13	155.91 (12)	C6—C5—C13—O3	179.07 (16)
C7—C4—C5—C13	−81.02 (15)	C4—C5—C13—O3	−4.1 (2)
C13—C5—C6—N1	−179.52 (13)	C6—C5—C13—O4	−0.8 (2)
C4—C5—C6—N1	3.74 (19)	C4—C5—C13—O4	176.03 (12)
C13—C5—C6—C16	−1.2 (2)	O3—C13—O4—C14	0.9 (3)
C4—C5—C6—C16	−177.95 (12)	C5—C13—O4—C14	−179.27 (15)
C2—N1—C6—C5	16.6 (2)	C13—O4—C14—C15	−175.54 (17)
C2—N1—C6—C16	−161.95 (13)	C5—C6—C16—C17	71.9 (2)
N3—C4—C7—C8	93.30 (16)	N1—C6—C16—C17	−109.73 (15)
C5—C4—C7—C8	−29.33 (19)	C6—C16—C17—O5	−163.22 (15)
N3—C4—C7—C12	−82.87 (15)	C6—C16—C17—O6	18.42 (19)
C5—C4—C7—C12	154.50 (12)	O5—C17—O6—C18	−3.9 (3)
C12—C7—C8—C9	0.8 (2)	C16—C17—O6—C18	174.44 (17)
C4—C7—C8—C9	−175.40 (15)	C17—O6—C18—C19	164.6 (2)
C7—C8—C9—C10	−0.2 (3)		

### Hydrogen-bond geometry (Å, °)

**Table:**

*D*—H···*A*	*D*—H	H···*A*	*D*···*A*	*D*—H···*A*
N1—H1···O1^i^	0.86	1.97	2.788 (2)	159
O2—H2···O3^ii^	0.82	1.95	2.772 (2)	177
N3—H3···O5^iii^	0.86	2.20	3.0014 (18)	155

Symmetry codes: (i) −*x*, −*y*+1, −*z*; (ii) −*x*+1, −*y*, −*z*+1; (iii) *x*, *y*−1, *z*.
